# Integrating Foundation Model Features into Graph Neural Network and Fusing Predictions with Standard Fine-Tuned Models for Histology Image Classification

**DOI:** 10.3390/bioengineering12121332

**Published:** 2025-12-06

**Authors:** Nematollah Saeidi, Nima Torbati, Ramona Woitek, Amirreza Mahbod

**Affiliations:** Research Center for Medical Image Analysis and Artificial Intelligence, Department of Medicine, Faculty of Medicine and Dentistry, Danube Private University, 3500 Krems an der Donau, Austria

**Keywords:** image classification, graph neural network, foundation model, medical image analysis, deep learning, computational pathology

## Abstract

Histopathological image classification using computational methods such as fine-tuned convolutional neural networks (CNNs) has gained significant attention in recent years. Graph neural networks (GNNs) have also emerged as strong alternatives, often employing CNNs or vision transformers (ViTs) as node feature extractors. However, as these models are usually pre-trained on small-scale natural image datasets, their performance in histopathology tasks can be limited. The introduction of foundation models trained on large-scale histopathological data now enables more effective feature extraction for GNNs. In this work, we integrate recently developed foundation models as feature extractors within a lightweight GNN and compare their performance with standard fine-tuned CNN and ViT models. Furthermore, we explore a prediction fusion approach that combines the outputs of the best-performing GNN and fine-tuned model to evaluate the benefits of complementary representations. Results demonstrate that GNNs utilizing foundation model features outperform those trained with CNN or ViT features and achieve performance comparable to standard fine-tuned CNN and ViT models. The highest overall performance is obtained with the proposed prediction fusion strategy. Evaluated on three publicly available datasets, the best fusion achieved F1-scores of 98.04%, 96.51%, and 98.28%, and balanced accuracies of 98.03%, 96.50%, and 97.50% on PanNuke, BACH, and BreakHis, respectively.

## 1. Introduction

Histopathology plays an important role in cancer diagnosis, prognosis, and treatment. Traditionally, histopathologists examine the microscopic structure of tissues using light microscopy to identify malignant features. Introduction of whole-slide digital scanners has transformed the field by enabling the generation of high-resolution digital slides. This digitization created opportunities for artificial intelligence-based methods in this field [1]. Automatic histological image analysis has become a critical tool in medical research and diagnostics, allowing for detailed examination of tissue structures at a microscopic level [2]. Image classification, a fundamental task in computational pathology, aims to assign diagnostic labels to these images or their subregions, such as distinguishing malignant from benign tissues [3]. Machine learning tools have emerged as powerful methods for various analysis tasks, such as regression, registration, and classification [4,5]. For histological image classification, various automated approaches have been proposed, ranging from classical machine learning to modern deep learning methods [6]. Convolutional neural networks (CNNs) [7], vision transformer (ViT)-based architectures [8], fused CNN–ViT models [9], and multimodal deep learning [10] are examples of such approaches. In addition to these, graph neural networks (GNNs) are also considered an alternative that can be utilized for medical and histological image classification tasks [11,12].

To apply GNNs to histology image classification, the first step is to construct a graph representation of the image, followed by the design of a graph-based architecture for classification [13]. Graph construction involves defining nodes, their embeddings, and the edges that connect them. Nodes can represent different histological entities, such as cell nuclei, image patches, or segmented regions (e.g., glands or tumors), while node embeddings are typically derived from features extracted by CNNs or ViTs [11]. Edges are generally established using spatial proximity or similarity measures to capture meaningful relationships between nodes [13]. Once the graph is constructed, GNN architectures can be employed for classification. Common building blocks include graph convolutional blocks [14], graph attention networks (GAT) [15], and GraphSAGE [16], which can be combined with pooling layers and fully connected or linear layers to perform the final classification. These models can be trained using standard loss functions such as categorical cross-entropy. More details and an overview of current trends in applying GNNs to medical and histological image analysis can be found in recent review articles [11,17].

In this study, we focus on graph node feature extraction. Many feature extraction methods have been proposed in the literature. Classical machine learning approaches typically rely on handcrafted or engineered features such as morphological and textural descriptors [18,19]. Due to the redundancy and computational cost of such features, several feature selection and dimensionality reduction techniques have also been proposed [20,21]. However, since extracting meaningful and informative features heavily depends on the specific application and dataset, handcrafted features may not optimally capture clinically relevant image characteristics, particularly in challenging cases [22].

With the advent of deep learning, and in particular, standard pre-trained CNNs, this domain has been revolutionized. CNN-based methods have become dominant due to their ability to automatically learn hierarchical and discriminative feature representations from images [23]. Pre-trained CNN architectures such as EfficientNet (V1 and V2) [24,25] and ResNet [26] have been widely used as feature extractors in both medical and non-medical image analysis tasks, including within GNN frameworks [27,28]. In addition to CNNs, Vision Transformer (ViT) models have also been recently explored as graph node feature extractors [11,29]. Owing to their ability to model long-range dependencies and capture global context through self-attention mechanisms, ViTs can provide highly informative features and, in some cases, outperform CNN-based features in specific image analysis tasks, particularly on large-scale datasets. While both CNNs and ViTs have demonstrated strong performance in many medical imaging tasks, they are usually pre-trained on natural image datasets (e.g., ImageNet [30]). The domain shift between natural and medical images can limit their ability to capture subtle, domain-specific histological patterns, which in turn may constrain their deployment in practical clinical settings [31]. Although various feature augmentation strategies, such as color jittering and deterministic or non-deterministic stain normalization, have been proposed to mitigate this issue [32,33], the domain shift and generalization challenges remain largely unsolved.

Recent advancements in pathology foundation models, specifically developed for histological image analysis, offer a promising alternative by providing robust and generalizable feature representations that can be adapted to specific tasks with minimal fine-tuning. Trained on large and diverse histology datasets, these models capture rich semantic and structural information, making them well-suited for feature extraction in complex histology domains. While they have been exploited for many downstream tasks in computational pathology, such as nuclei segmentation [34,35], histology image retrieval [28,36], and histology image segmentation [37,38], these foundation models can also be integrated into GNN frameworks to represent graph node features more effectively.

In this work, we leverage three recently developed pathology foundation models (CONCH [39], UNI [38], and UNI2 [38,40]) within a lightweight GNN as graph node feature extractors for histological image classification. We evaluate their classification performance against well-known pre-trained CNNs and ViT models (VGG19 [41], DenseNet201 [42], EfficientNetV2S [25], and ViT-Base [43]) on three publicly available benchmark datasets: PanNuke [44], BACH [45], and BreakHis [46]. Furthermore, we fuse the predictions of the best-performing GNN trained with foundation model features with those of the best-performing standard fine-tuned model to further improve classification performance.

In summary, the main contributions of this study can be listed as follows:

- We utilize pathology foundation models within a lightweight GNN for histological image classification.
- We compare the results of the proposed approach with standard fine-tuned CNN and ViT models, as well as GNNs using CNN and ViT features as node embeddings.
- We propose a fusion approach based on weighted averaging that combines the results from the best GNN models with the best standard fine-tuned models to further improve classification performance.
- We make our implementation publicly available to ensure reproducibility and facilitate future research.

The remainder of this manuscript is organized as follows: Section 2 provides a description of the datasets used in this study, an overview of the baseline CNN, ViT, and pathology foundation models, the architecture of the developed lightweight GNN model, the evaluation metrics utilized, the fusion and the implementation details. Section 3 presents and discusses the experimental results, and Section 4 concludes the work.

## 2. Materials and Methods

We propose a lightweight GNN-based model that leverages foundation model features as graph node representations to perform classification on histological images. We compare the performance of our proposed method with a number of baseline fine-tuned CNN and ViT models, as well as baseline GNN models that utilize standard node features extracted from either CNN-based or ViT-based pre-trained models.

### 2.1. Datasets

To conduct experiments, we use three datasets: PanNuke [44], BACH [45], and BreakHis [46]. All datasets contain samples extracted from whole-slide hematoxylin and eosin (H&E)-stained images. A brief description of each dataset is provided below, and further details can be found in the respective original studies.

The PanNuke dataset contains 7901 image patches, each with a size of 256 × 256 pixels, captured at 40× magnification, and extracted from 19 different organs. The original dataset provides nuclei instance segmentation and classification masks with five types of nuclei, namely neoplastic tumor cells, inflammatory cells, connective tissue cells, dead cells, and epithelial cells. Since our objective is to perform a classification task, we construct a binary classification dataset by labeling samples from a subset of the PanNuke dataset as either malignant or benign, considering only images that contain at least one cell. Following the procedure proposed in [47], this results in a dataset comprising 2873 malignant and 3367 benign images.

The second dataset used in this study is the BACH dataset. It consists of 400 image patches of breast cancer tissue. Each image has a size of 2048 × 1536 pixels and was captured at 20× magnification. The images are labeled into four distinct classes, namely benign, in situ carcinoma, invasive carcinoma, and normal, with 100 images in each class.

The third and final dataset used in this study is the BreakHis dataset, which contains breast histological images at different magnification factors, divided into two classes: malignant and benign. The dataset comprises a total of 7909 images. However, in this work, we used only the images acquired at a magnification factor of 400× (corresponding to objective lens 40×), which is the common magnification used in routine pathology. This subset includes 338 benign images and 982 malignant images. All images are provided at a fixed size of 700×460 pixels.

**Figure 1:**
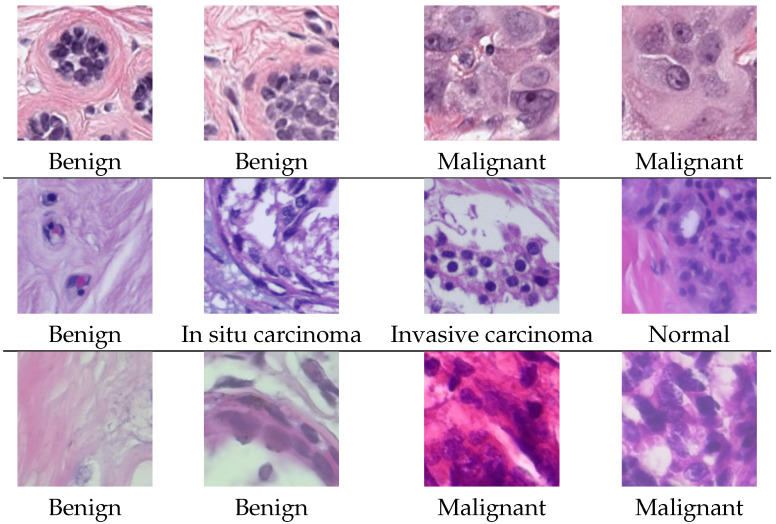
Example images from the PanNuke dataset (**first row**), the BACH dataset (**second row**) and the BreakHis dataset (**third row**).

Example images from each dataset are shown in Figure 1.

### 2.2. Baseline Fine-Tuned Models

To establish a baseline for comparison, we use four pre-trained models in this study, namely VGG19 [41], DenseNet201 [42], EfficientNetV2S [25], and ViT-Base models [43].

VGG is one of the most well-known CNN architectures and has been widely applied to both medical and non-medical image classification tasks [48]. It primarily consists of convolutional layers, max-pooling layers, and fully connected layers. Several versions of the model exist, varying in depth. In this study, we use the pre-trained VGG19 variant. The model was pre-trained on the ImageNet dataset, which contains 1.2 million natural images across 1000 classes.

Another widely used CNN model employed in this study as a baseline is DenseNet [42]. In addition to standard building blocks such as convolutional layers, DenseNet uses the concept of skip connections by connecting each layer to every other layer in a feed-forward fashion. This design improves gradient flow and enhances model performance. Similar to VGG19, DenseNet has multiple depth variants. In this work, we use DenseNet201, pre-trained on the ImageNet dataset.

The third CNN baseline model used in this study is EfficientNetV2S. EfficientNetV2 is the second generation of the EfficientNet family [24], designed to improve training speed, parameter efficiency, and overall performance. It achieves this by combining mobile inverted bottleneck (MB) convolutional blocks with Fused-MB convolutional blocks, along with techniques such as a non-uniform scaling strategy and progressive learning. In this work, we use the EfficientNetV2S variant, which is the fastest model in the EfficientNetV2 family and is also pre-trained on the ImageNet dataset.

Besides these CNN models, we also employ a ViT-based model. In contrast to CNNs, which process images using localized convolutional filters, a ViT model represents images as flattened patch embeddings (e.g., patches of size 16 × 16 pixels) and processes them through a transformer encoder that applies self-attention to capture global relationships among all patches. For classification tasks, the transformer encoder is typically followed by a multi-layer perceptron (MLP) head to generate the final predictions. Similar to CNNs, ViTs come in different architectures such as ViT-Tiny and ViT-Small; in this work, we employ the commonly used ViT-Base (ViT-B/16) model pre-trained on the ImageNet dataset.

Although several other pre-trained CNN and ViT models exist, we explicitly selected VGG19 and DenseNet201 as they are well-established benchmark models in numerous classification studies involving datasets of various sizes, different numbers of classes, and varying levels of complexity [49,50,51]. EfficientNetV2-S, on the other hand, is a more recently developed model that offers excellent classification performance while remaining computationally efficient [24,52]. We also included ViT-Base as a representative transformer-based model, as it provides superior performance compared to smaller variants such as ViT-Tiny and ViT-Small, while being less computationally expensive than larger models such as ViT-Large and ViT-Huge [43].

To adapt these models for the histological image classification tasks on our employed datasets, we replaced the original fully connected layers with a single linear layer, where the number of output nodes corresponds to the number of classes in each dataset (two for PanNuke and BreakHis, and four for BACH). The input images were resized to a fixed resolution of 224 × 224 pixels and normalized using the mean and standard deviation of the ImageNet dataset. In addition, standard data augmentation techniques, including random horizontal and vertical flipping as well as random rotations, were applied during fine-tuning.

### 2.3. GNN Model

**Figure 2:**
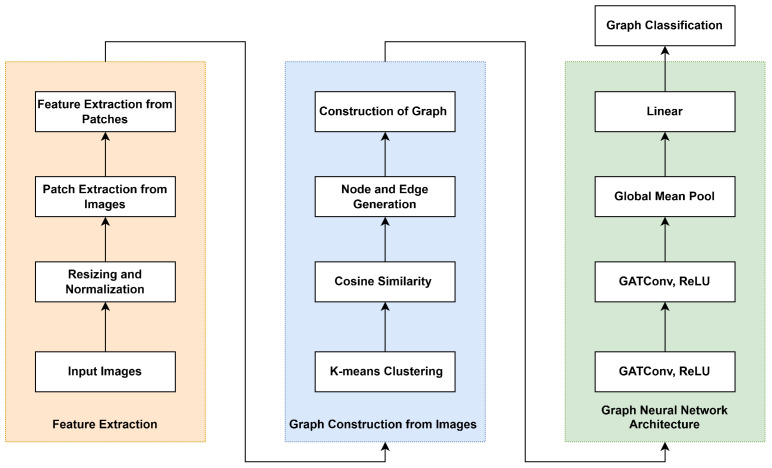
General workflow of the proposed approach.

In this work, we develop a lightweight GNN as illustrated in Figure 2. The proposed method consists of three main components, namely feature extraction from image patches (orange section), graph construction (blue section), and the GNN architecture (green section).

#### 2.3.1. Feature Extraction

The first step prior to feature extraction is resizing all images to a fixed size of 224 × 224 pixels and normalizing them using the mean and standard deviation of the ImageNet dataset. Non-overlapping patches are then extracted from the input images. These patches are subsequently passed to the pre-trained models, and the resulting embeddings are used for graph construction. For pre-trained models, we employ the same CNNs and ViT-Base as baseline models, along with three pathology foundation models: CONCH [39], UNI [38], and UNI-2 [38,40]. As fine-tuning these models significantly increases computational cost, all foundation models are used in their original pre-trained form without further domain adaptation or fine-tuning, similar to a number of other studies that also employ these models in a frozen form [28,53].

Although several medical and non-medical foundation models have been developed and proposed in recent years, we selected pathology-specific foundation models that have demonstrated excellent performance in previous studies. For instance, the UNI and CONCH models have shown superior performance compared to a number of other foundation models such as MedCLIP, BioMedCLIP, and Virchow in [28]. Similarly, UNI has also outperformed other pathology foundation models such as CTransPath and Phikon in [54]. UNI-2 represents an improved version of UNI, trained on a larger and more diverse dataset, and has exhibited better performance than the original UNI model [55]. A brief description of the selected foundation models is provided below.

CONCH is a vision–language pathology foundation model trained on 1.17 million histological image–text pairs using the CLIP paradigm [56], employing a vision encoder (ViT-B/16) and a GPT-style text encoder. UNI is a vision foundation model trained on more than 100 million histological image patches using the DINOv2 self-supervised learning approach [57]. Image embeddings are extracted from the UNI encoder, which is based on the ViT-L/16 architecture. We also utilize UNI-2, an extension of the original UNI model. UNI-2 is pre-trained on more than 200 million histological images using a ViT-H/14 vision encoder, making it one of the largest foundation models available for computational pathology tasks. Unlike CONCH and UNI, which extract 16 × 16 image patches, UNI-2 uses 14 × 14 patches due to its different vision encoder architecture.

The patch size, the number of extracted patches, and the size of the extracted features from each model (both standard and foundation models) are reported in the third, fourth, and fifth columns of Table 1, respectively. It should be noted that standard CNN models require larger patch sizes due to the technical limitation that smaller images cannot be used for feature extraction from pre-trained CNNs.

#### 2.3.2. Graph Construction

The extracted features from image patches serve as the basis for graph construction. As the main focus of this study is to investigate the effect of integrating pathology foundation model features with a GNN, rather than developing or using advanced and computationally expensive graph construction methods, we design a lightweight GNN, as illustrated in Figure 2. For ViT-Base and pathology foundation models, we apply the k-means clustering algorithm to partition the features into a predefined number of clusters, set by default to 100 to keep the model lightweight. For clustering, we use a fixed random seed to ensure reproducibility. The resulting cluster centroids, which capture both the spatial and semantic characteristics of the image patches, are designated as the nodes of the graph. The input and output data sizes of the clustering algorithm across different models are summarized in the fifth and sixth columns of Table 1.

To establish connectivity between the nodes, we compute a cosine similarity matrix over all pairs of cluster centroids, quantifying their pairwise similarity. Edges are then defined based on this matrix. Specifically, for each pair of distinct clusters, if their similarity exceeds an empirically derived threshold of 0.6, bidirectional edges are added to the edge list. This lightweight graph construction strategy effectively condenses high-dimensional patch features into a compact and structured representation.

#### 2.3.3. GNN Architecture

We utilize the graph attention network (GAT) [15] as the core building block to operate on the graph representations derived in the previous stage. GAT introduces an attention mechanism that enables nodes to weigh the importance of their neighbors and allows the network to focus on the most relevant connections in the graph. Our designed GNN model consists of two GATConv layers. These layers aggregate information from neighboring nodes based on learned attention weights, updating node representations in a way that preserves both feature and structural information. The first GATConv layer has four attention heads, each producing eight features, resulting in a combined output of 32 features per node, followed by a ReLU layer. The second GATConv layer is also equipped with four attention heads, each generating four output features per head, leading to a 16-dimensional representation per node, again followed by a ReLU layer. The output of the second ReLU layer is connected to a global mean pooling and a linear layer that maps the graph representation to the number of classes (two for the PanNuke and BreakHis datasets, and four for the BACH dataset). The linear projection enables the model to perform the final classification task by predicting the class label associated with each image.

It should be noted that our implementation choices, such as using two GATConv layers and applying a mean pooling layer to average the feature vectors of all nodes in the graph, were inspired by previous CNN- and GNN-based studies [58,59,60].

### 2.4. Evaluation

To evaluate the performance of the models for both binary and multi-class histological image classification, we employ two well-known evaluation metrics, namely F1-score (macro average for multi-classes) and balanced accuracy, as follows:

(1) $$F1=2\times \frac{\mathrm{Precision}\times \mathrm{Recall}}{\mathrm{Precision}+\mathrm{Recall}}$$

where precision and recall can be derived from the following formulas:

(2) $$\mathrm{Precision}=\frac{TP}{TP+FP}\;\mathrm{Recall}=\frac{TP}{TP+FN}$$

TP, FP, and FN represent true positives, false positives, and false negatives, respectively. For multi-class datasets, the macro F1-score can be derived as follows:

(3) $${F1}^{\mathrm{macro}}=\frac{1}{C}\sum_{i=1}^{C}{F1}^{\left(i\right)}$$

where *C* represents the number of classes.

Balanced accuracy can be derived from the following formula:

(4) $$\mathrm{Balanced}\;\mathrm{Accuracy}=\frac{1}{C}\sum_{i=1}^{C}\frac{TP_{i}}{TP_{i}+FN_{i}}$$

where again *C*, TP, and FN represent the number of classes, true positives, and false negatives, respectively.

To report the results, we calculate the mean and standard deviation of these metrics over 5-fold cross-validation to report the results. Besides reporting results in our experiments based on these two metrics, we also provide additional evaluation metrics, including precision, recall, sensitivity, specificity, area under the receiver operating characteristic curve (AUC), and Matthews Correlation Coefficient (MCC), as suggested in [61]. These metrics are reported for our best results and are available as Supplementary Materials in our GitHub repository (Tables S1–S3).

### 2.5. Fusion and Implementation Details

To perform fusion, we used weighted averaging as follows:

(5) $$P_{\mathrm{fusion}}=w\cdot P_{A}+\left(1-w\right)\cdot P_{B}$$

where $P_{\mathrm{fusion}}$ denotes the fused prediction obtained by ensembling, $P_{A}$ and $P_{B}$ represent the predictions of *Model A* and *Model B*, respectively, and w∈[0,1] is the weighting factor assigned to *Model A* (with 1−w assigned to *Model B*). We empirically selected the optimal *w* within the range [0,1] using a step size of 0.1.

For the implementation of the standard baseline models as well as graph construction, we use the PyTorch (version 2.5.1) deep learning framework. To extract features from the CONCH, UNI, and UNI2 foundation models, we follow the code and instructions provided on the respective GitHub pages at https://github.com/mahmoodlab/UNI (accessed on 3 December 2025) and https://github.com/mahmoodlab/CONCH (accessed on 3 December 2025).

All experiments are conducted using 5-fold cross-validation, and the results are reported as the average and standard deviation across the folds. For the standard baseline models, we used the Adam optimizer with a learning rate of $1\times 10^{-4}$, trained for 300 epochs with a batch size of 32, and a fixed seed point for reproducibility. The graph-based models were similarly optimized with Adam and a learning rate of $1\times 10^{-4}$, but trained for 300 epochs with a batch size of 32 and the same fixed seed point, using the cross-entropy loss function across all experiments.

As noted earlier, we used a fixed cluster size of 100 for the GNN-based experiments employing ViT, CONCH, UNI, and UNI2 feature extractors. Alternative cluster sizes were also evaluated, but the results did not vary substantially (refer to the Results section). For the GNN-based experiments with VGG19, EfficientNetV2S, and DenseNet201, we used 49 clusters, corresponding to the number of generated patches, since larger numbers could not be applied due to this constraint (see the fifth column in Table 1).

Experiments were run on a single workstation equipped with an Intel Xeon(R) Gold 6326 CPU running at 2.90 GHz, 47 GB of RAM, and an NVIDIA A40-16Q GPU with 16 GB of available memory. The code developed to generate and reproduce the results is publicly available in our GitHub repository at https://github.com/nematollahsaeidi/his_img_GNN_classification (accessed on 3 December 2025).

## 3. Results & Discussion

The classification results from our experiments are presented in Table 2, Table 3 and Table 4 for the PanNuke, BACH, and BreakHis datasets, respectively. The tables are divided into four parts: the first part shows the performance of fine-tuning standard models; the second part reports the performance of GNN models with standard feature extraction using pre-trained CNNs or ViT; the third part presents the performance of GNNs with pathology foundation model feature extraction; and the fourth part shows the performance of prediction fusion between the best GNN models (GNN-UNI or GNN-UNI2) and the overall best-performing standard CNN models (EfficientNetV2S and DensNet201).

As shown in the tables, DenseNet201 and EfficientNetV2-S achieve the best overall performance among the standard fine-tuned models. For GNNs trained with features from standard models, GNN-ViT outperforms GNN-VGG19, GNN-DenseNet201, and GNN-EfficientNetV2-S by a considerable margin, with the exception of the BreakHis dataset, where GNN-DenseNet201 delivers slightly better performance than GNN-ViT. Nevertheless, the fine-tuned CNN and ViT models consistently outperform GNNs trained on pre-trained CNN or ViT features.

In contrast, GNNs trained with foundation model features outperform baseline GNNs in most cases. Among the evaluated foundation models, CONCH yields the lowest performance, while UNI and UNI-2 demonstrate competitive results.

Among the foundation models, UNI and UNI2 show strong performance on both the multi-organ (PanNuke) and single-organ (BACH and BreakHist) datasets. While CONCH performs comparably to the UNI family on the PanNuke and BreakHist datasets, its performance decreases notably on BACH. This may be due to the larger original image size in BACH relative to the other datasets, highlighting CONCH’s sensitivity to image resolution.

Fusion strategies combining the best foundation models (UNI or UNI-2) with the top-performing fine-tuned models (EfficientNetV2-S and DenseNet201) yield slight performance improvements, highlighting the potential of leveraging complementary information through model fusion. Among the fused models, the combination of GNN-UNI2 and DenseNet201 achieves the best overall performance.

**Figure 3:**
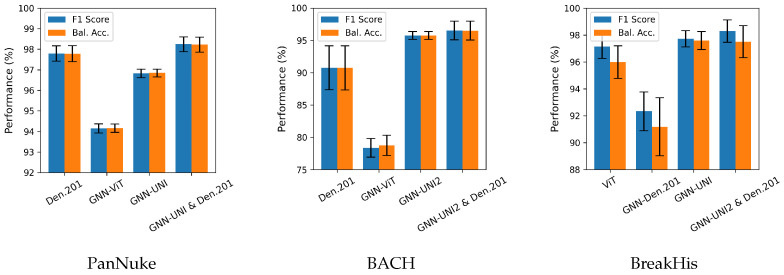
Visual comparison of the best-performing methods on the PanNuke, BACH, and BreakHis datasets. For each dataset, the performance of the best models from each part of the corresponding tables (Table 2, Table 3 and Table 4) is shown.

**Figure 4:**
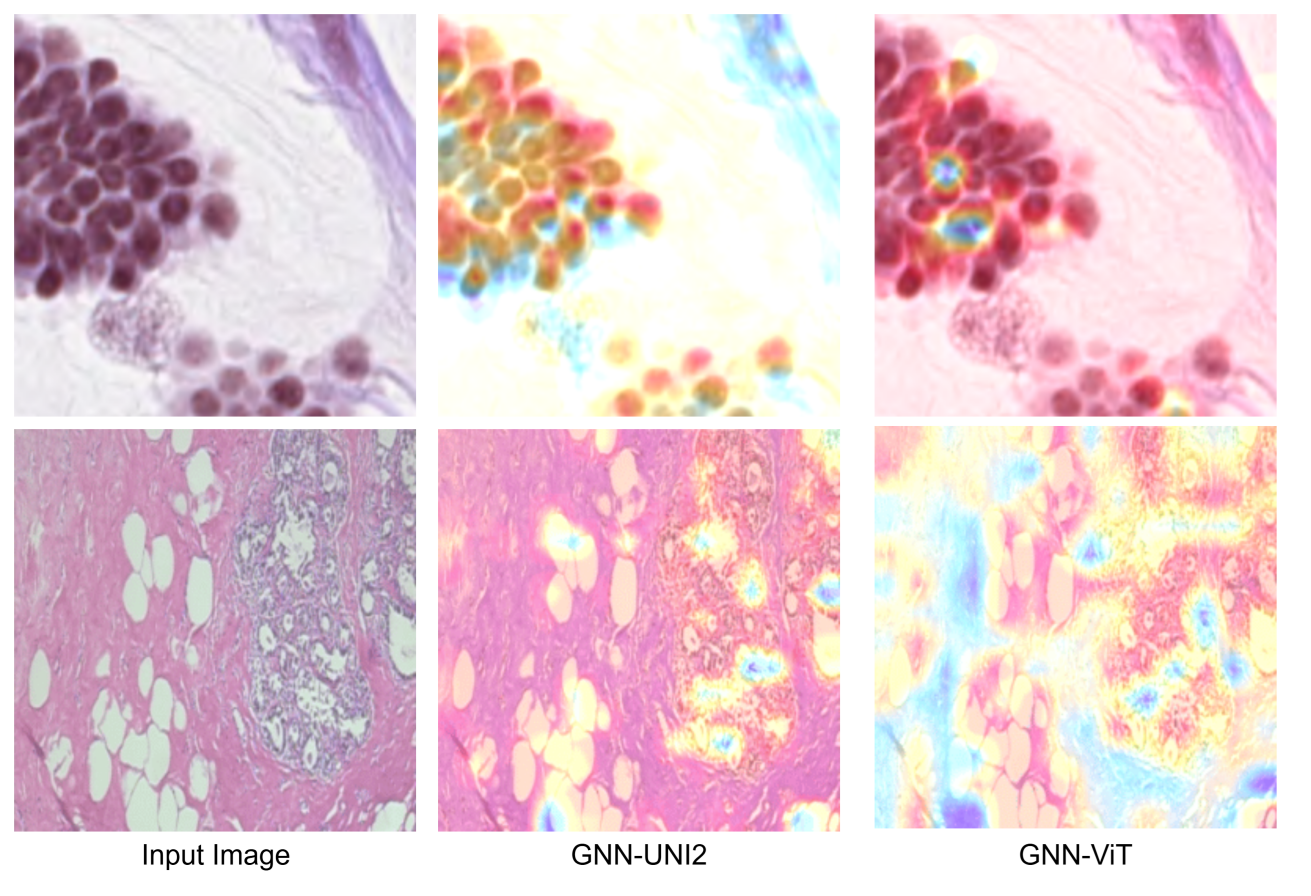
Attention heatmaps generated using the Grad-CAM method for two example images are shown for the GNN-UNI2 and GNN-ViT models. In both cases, GNN-UNI2 correctly classified the images, whereas GNN-ViT produced incorrect predictions. As illustrated, GNN-UNI2 focuses more strongly on clinically relevant regions of the tissue compared to GNN-ViT.

For better visualization, bar charts of the best-performing methods from each part of the results tables are shown in Figure 3. Additionally, in Figure 4, we present the attention heatmaps of example images using the Grad-CAM method [62] for the GNN-UNI2 (the best overall GNN model with foundation model features) and GNN-ViT models (the best overall GNN model with standard model features). In both cases, GNN-UNI2 correctly classified the images, whereas GNN-ViT produced incorrect predictions. As illustrated, GNN-UNI2 focuses more strongly on clinically relevant regions of the tissue compared to GNN-ViT.

We also calculated the 95 percent confidence intervals for the best GNN-based models using the t values from the Student’s t distribution, and we report the results in Table 5. The GNN-UNI2 model consistently outperforms GNN-ViT on the BACH and BreakHis datasets, as indicated by the non-overlapping 95 percent confidence intervals, whereas for PanNuke, the intervals overlap, suggesting comparable performance.

Besides the main results presented in the previous tables, we conduct a number of ablation studies. First, we evaluate the impact of the number of clusters on classification performance. This analysis is performed using the best GNN model (GNN-UNI2) with different numbers of clusters (10, 30, 50, and 100) on the PanNuke dataset. As the results in Table 6 indicate, varying the number of clusters does not drastically affect performance; however, the empirically chosen value of 100 clusters yields slightly improved results.

We also investigate the effect of the similarity threshold value on classification performance, and the results based on the GNN-UNI2 model are reported in Table 7. As the results show, the chosen threshold value of 0.6 delivers superior performance compared to other tested thresholds.

To assess the performance of the weighted averaging strategy, we compare it with other approaches, namely simple averaging and meta-learning using logistic regression and a two-layer neural network, and report the results in Table 8. As shown in the table, the weighted averaging method achieves slightly superior performance while being computationally less expensive compared to the two meta-learners.

To estimate the computational cost, we measure both the training and testing times of different models, as well as GPU memory consumption. The results for the BreakHis dataset are reported in Table 9 and Table 10 as representative examples. As shown in Table 9, the training time of GNN-based approaches is considerably lower than that of fine-tuning methods; however, fine-tuned models achieve faster inference. In the case of GNN models, most of the training and testing time is consumed by graph construction rather than by the GNN model training or inference itself. In fact, when excluding the graph construction time, GNN models are significantly faster than fine-tuned models during inference.

We observed a similar pattern in GPU memory consumption. As the quantitative results in Table 10 show, training GNN-based models required much less memory compared to standard fine-tuned CNN or ViT models, although graph construction consumed a substantial amount of GPU memory.

Despite the promising results reported in this study, several limitations should be acknowledged.

First, although three well-established benchmark datasets were used for training and evaluation, using larger-scale datasets or extending the approach to whole-slide image classification would allow for a more comprehensive assessment.

Second, only a specific set of foundation and baseline models was considered. Future work could investigate a wider range of both medical and non-medical foundation models, as well as additional baseline architectures. The models included in this study were deliberately chosen due to their strong performance in previous computational pathology and computer vision tasks [28,38,39] and because the baselines are widely adopted in several related studies for medical image classification [45,63].

Third, prediction fusion was limited to a weighted averaging strategy. Alternative ensemble methods may provide further improvements in classification performance and should be explored in subsequent work.

Fourth, although the datasets used in this study capture only the static characteristics of sampled tissue, future work could explore applying the proposed method to datasets with longitudinal information, enabling the analysis of tumor region dynamics over time [64].

Finally, the main computational bottleneck of GNN-based approaches lies in graph construction rather than in training or inference. Future research should therefore explore strategies such as batch-based parallel computation for preprocessing and graph generation to reduce runtime.

## 4. Conclusions

Deep learning has transformed image analysis, yet most feature extractors, such as standard CNNs and ViTs used in computational pathology, still rely on models trained on natural images. This mismatch limits their ability to capture the fine-grained morphological cues that drive clinical decision-making. Pathology-specific foundation models represent an important step forward because they are trained on large and diverse histological data and therefore provide features that are substantially more aligned with the domain. Our work builds on this emerging trend by examining how these specialized representations behave within graph-based learning pipelines.

We systematically investigated the integration of foundation model features with graph-based neural networks for histological image classification. Using three benchmark datasets, we demonstrated that pathology-specific foundation models enhance GNN performance compared to conventional CNN- or ViT-based feature extractors, while also providing competitive results against standard fine-tuned baseline models. Furthermore, we showed that fusing predictions from foundation model feature-based GNNs and fine-tuned CNNs yields slight performance gains, underscoring the complementary nature of these representations. Although graph construction introduces computational overhead, our analysis confirmed the efficiency of the proposed pipelines relative to purely fine-tuning approaches when considering model training and inference times alone.

Looking ahead, this line of work offers several promising extensions. Future work can focus on extending this framework to larger and more diverse datasets, evaluating additional baseline and foundation models, application to whole-slide level prediction, exploring more advanced ensemble strategies, and further optimizing graph construction. Overall, our findings highlight the promise of combining foundation model representations with graph-based learning to advance the state of computational pathology.

## Figures and Tables

**Table 1:** Image, patch, and clustering sizes for the algorithm. Den.201: DenseNet201, Eff.V2S: EfficientNetV2S, ViT-B: Vision Transformer-Base.

Model	Image Size	Patch Size	#Patches	Input Size (Clustering)	Output Size (Clustering)
VGG19	224 × 224	32 × 32	7 × 7	49 × 512	49 × 512
Den.201	224 × 224	32 × 32	7 × 7	49 × 1920	49 × 1920
Eff.V2S	224 × 224	32 × 32	7 × 7	49 × 1280	49 × 1280
ViT-B	224 × 224	16 × 16	14 × 14	196 × 768	100 × 768
CONCH	224 × 224	16 × 16	14 × 14	196 × 512	100 × 512
UNI	224 × 224	16 × 16	7 × 7	196 × 1024	100 × 1024
UNI2	224 × 224	14 × 14	16 × 16	256 × 1536	100 × 1536

**Table 2:** Classification performance on the PanNuke dataset. Bal. Acc.: Balanced Accuracy, Den.201: DenseNet201, Eff.V2S: EfficientNetV2S, ViT: Vision Transformer-Base. The bold results indicate the best performance for each metric.

Model	F1-Score (%)	Bal. Acc. (%)
VGG19	96.26 (±0.17)	96.28 (±0.17)
Den.201	97.79 (±0.37)	97.78 (±0.39)
Eff.V2S	97.58 (±0.33)	97.58 (±0.30)
ViT	97.55 (±0.57)	97.54 (±0.59)
GNN-VGG19	90.51 (±0.38)	90.55 (±0.38)
GNN-Den.201	90.14 (±0.59)	90.16 (±0.57)
GNN-Eff.V2S	87.78 (±0.26)	87.85 (±0.27)
GNN-ViT	94.15 (±0.22)	94.16 (±0.20)
GNN-CONCH	89.30 (±0.78)	89.34 (±0.78)
GNN-UNI	96.82 (±0.21)	96.84 (±0.19)
GNN-UNI2	93.07 (±0.37)	93.00 (±0.38)
GNN-UNI & Eff.V2S	98.17 (±0.16)	98.14 (±0.16)
GNN-UNI2 & Eff.V2S	97.95 (±0.29)	97.94 (±0.28)
GNN-UNI & Den.201	**98.24 (±0.36)**	**98.22 (±0.37)**
GNN-UNI2 & Den.201	98.04 (±0.36)	98.03 (±0.36)

**Table 3:** Classification performance on the BACH dataset. Bal. Acc.: Balanced Accuracy, Den.201: DenseNet201, Eff.V2S: EfficientNetV2S, ViT: Vision Transformer-Base. The bold results indicate the best performance for each metric.

Model	F1-Score (%)	Bal. Acc. (%)
VGG19	81.91 (±3.91)	82.00 (±3.76)
Den.201	90.76 (±3.39)	90.75 (±3.41)
Eff.V2S	85.40 (±1.92)	85.50 (±1.87)
ViT	84.35 (±1.06)	84.50 (±1.00)
GNN-VGG19	59.88 (±4.26)	60.25 (±4.36)
GNN-Den.201	66.53 (±2.33)	67.00 (±2.18)
GNN-Eff.V2S	58.11 (±1.20)	57.75 (±1.46)
GNN-ViT	78.37 (±1.44)	78.75 (±1.58)
GNN-CONCH	69.61 (±4.17)	69.75 (±4.43)
GNN-UNI	91.95 (±1.71)	92.00 (±1.70)
GNN-UNI2	95.77 (±0.61)	95.75 (±0.61)
GNN-UNI & Eff.V2S	92.96 (±1.01)	93.00 (±0.99)
GNN-UNI2 & Eff.V2S	96.26 (±0.79)	96.25 (±0.79)
GNN-UNI & Den.201	93.22 (±1.07)	93.24 (±0.99)
GNN-UNI2 & Den.201	**96.51 (±1.44)**	**96.50 (±1.45)**

**Table 4:** Classification performance on the BreakHis dataset. Bal. Acc.: Balanced Accuracy, Den.201: DenseNet201, Eff.V2S: EfficientNetV2S, ViT: Vision Transformer-Base. The bold results indicate the best performance for each metric.

Model	F1-Score (%)	Bal. Acc. (%)
VGG19	96.16 (±0.72)	96.47 (±0.75)
Den.201	96.56 (±0.91)	95.77 (±1.38)
Eff.V2S	96.56 (±0.40)	95.58 (±0.67)
ViT	97.15 (±0.90)	95.98 (±1.22)
GNN-VGG19	87.88 (±0.80)	86.53 (±1.43)
GNN-Den.201	92.33 (±1.44)	91.18 (±2.15)
GNN-Eff.V2S	83.63 (±2.13)	80.96 (±1.89)
GNN-ViT	91.24 (±1.03)	90.14 (±1.23)
GNN-CONCH	91.40 (±1.34)	91.00 (±1.31)
GNN-UNI	97.71 (±0.60)	97.58 (±0.67)
GNN-UNI2	95.39 (±0.60)	94.87 (±0.78)
GNN-UNI & Eff.V2S	98.09 (±0.59)	**97.68 (±0.85)**
GNN-UNI2 & Eff.V2S	97.97 (±0.72)	97.05 (±1.03)
GNN-UNI & Den.201	98.09 (±0.59)	**97.68 (±0.85)**
GNN-UNI2 & Den.201	**98.28 (±0.83)**	97.50 (±1.19)

**Table 5:** Performance of the best GNN-based approaches using UNI-2 foundation model features and ViT features with 95% confidence intervals (t distribution). Values are reported as mean ± standard deviation using 5-fold cross-validation.

Dataset	Model	Mean ± Std	CI 95% (t-Dist.)
BACH	GNN-UNI2	F1-Score: 95.77 ± 0.61	[95.01, 96.53]
		Bal. Acc: 95.75 ± 0.61	[94.99, 96.51]
	GNN-ViT	F1-Score: 78.37 ± 1.44	[76.58, 80.16]
		Bal. Acc: 78.75 ± 1.58	[76.79, 80.71]
PanNuke	GNN-UNI2	F1-Score: 93.07 ± 0.37	[92.61, 93.53]
		Bal. Acc: 93.00 ± 0.38	[92.53, 93.47]
	GNN-ViT	F1-Score: 94.15 ± 0.22	[93.88, 94.42]
		Bal. Acc: 94.16 ± 0.20	[93.91, 94.41]
BreakHis	GNN-UNI2	F1-Score: 95.39 ± 0.60	[94.65, 96.14]
		Bal. Acc: 94.87 ± 0.78	[93.90, 95.84]
	GNN-ViT	F1-Score: 91.24 ± 1.03	[89.96, 92.52]
		Bal. Acc: 90.14 ± 1.23	[88.61, 91.67]

**Table 6:** Classification performance of the GNN-UNI2 model with varying numbers of clusters on the PanNuke dataset. Bal. Acc.: Balanced Accuracy. The bold results indicate the best performance for each metric.

# Clusters	F1-Score (%)	Bal. Acc. (%)
10	92.51	92.47
30	92.88	92.84
50	92.78	92.72
100	**93.07**	**93.00**

**Table 7:** Classification performance of the GNN-UNI2 model with different similarity threshold values on the PanNuke dataset. Bal. Acc.: Balanced Accuracy. The bold results indicate the best performance for each metric.

Similarity Threshold	F1-Score (%)	Bal. Acc. (%)
0.2	91.74	91.68
0.4	92.84	92.78
0.6	**93.07**	**93.00**
0.8	92.91	92.85

**Table 8:** Classification performance of different ensembling approaches for GNN-UNI and DenseNet201 on the PanNuke dataset. The bold results indicate the best performance for each metric.

Ensembling	F1-Score (%)	Bal. Acc. (%)
Logistic Regression	98.15 (±0.74)	98.21 (±0.72)
2-layer Neural Network	97.99 (±0.57)	98.06 (±0.54)
Simple Average	98.19 (±0.16)	98.16 (±0.17)
Weighted Average	**98.24 (±0.36)**	**98.22 (±0.37)**

**Table 9:** Comparison of training and testing times across models on the BreakHis dataset. The reported values correspond to the average training time per cross-validation fold (in seconds) and the average testing time per image (in milliseconds).

Model	Train (s)			Test (ms)		
	Graph	Train	Total	Graph	Test	Total
VGG19	–	1880.99	1880.99	–	2.30	2.30
Den.201	–	3116.85	3116.85	–	2.80	2.80
Eff.V2S	–	2089.28	2089.28	–	2.00	2.00
ViT	–	3597.64	3597.64	–	3.500	3.50
GNN-VGG19	598.51	107.03	705.54	372.60	0.10	372.70
GNN-Den.201	744.10	119.95	864.05	459.10	0.20	459.30
GNN-Eff.V2S	679.39	134.88	814.27	424.80	0.20	425.00
GNN-ViT	1035.26	139.84	1175.10	644.60	0.20	644.80
GNN-Swin	1019.09	122.21	1141.30	627.70	0.20	627.90
GNN-CONCH	889.69	129.62	1019.31	540.00	0.20	540.20
GNN-UNI2	1941.12	118.89	2060.01	1193.30	0.20	1193.50
GNN-UNI	1423.49	121.18	1544.67	870.30	0.20	870.50

**Table 10:** Comparison of GPU memory consumption across models on the BreakHis dataset. The reported values correspond to the GPU memory during graph construction (in MB), during GNN/CNN/ViT training (in MB), and total memory (in MB).

Model	Graph (MB)	Train (MB)	Total (MB)
VGG19	–	1526.75	1526.75
Den.201	–	3473.20	3473.20
Eff.V2S	–	2755.62	2755.62
ViT	–	2986.85	2986.85
GNN-VGG19	548.42	63.84	612.26
GNN-Den.201	77.39	55.18	132.57
GNN-Eff.V2S	83.56	70.30	153.86
GNN-ViT	338.35	36.55	374.90
GNN-Swin	755.49	234.33	989.82
GNN-CONCH	1517.90	208.10	1726.99
GNN-UNI2	2631.94	43.84	2675.78
GNN-UNI	1166.32	44.53	1210.85

## Data Availability

The datasets used in this study are publicly available from previously published papers [44,45,46]. The code developed for this study is available on GitHub, with the corresponding link provided in the manuscript.

## Supplementary Materials

The following supporting information can be downloaded at: https://www.mdpi.com/article/10.3390/bioengineering12121332/s1, Table S1: Classification performance on the PanNuke dataset based on F1-score, balanced accuracy, precision, recall, sensitivity, specificity, area under the receiver operating characteristic curve (AUC), and Matthews Correlation Coefficient (MCC); Table S2: Classification performance on the BACH dataset based on F1-score, balanced accuracy, precision, recall, sensitivity, specificity, area under the receiver operating characteristic curve (AUC), and Matthews Correlation Coefficient (MCC); Table S3: Classification performance on the BreakHis dataset based on F1-score, balanced accuracy, precision, recall, sensitivity, specificity, area under the receiver operating characteristic curve (AUC), and Matthews Correlation Coefficient (MCC).

## Abbreviations

The following abbreviations are used in this manuscript:

GNN	Graph Neural Network
CNN	Convolutional Neural Network
ViT	Vision Transformer
GAT	Graph Attention Network
MB	Mobile Inverted Bottleneck
MLP	Multi-Layer Perceptron
Bal. Acc.	Balanced Accuracy
Den.201	DenseNet201
Eff.V2S	EfficientNetV2S
