# Supplementary material for: Integrating Foundation Model Features into Graph Neural Network and Fusing Predictions with Standard Fine-Tuned Models for Histology Image Classification
Source: Bioengineering (Basel). 2025 Dec 6;12(12):1332. doi: 10.3390/bioengineering12121332 (PMC12729508; doi:10.3390/bioengineering12121332)
Supplement: Supplementary file 1 [file bioengineering-12-01332-s001.zip › bioengineering-3954603-supplementary.pdf]

Integrating Foundation Model Features into Graph Neural Network and Fusing Predictions with Standard Fine-Tuned Models for Histology Image Classification

supplementary material

S1: Classification performance on the PanNuke dataset based on F1-score, balanced accuracy, precision, recall, sensitivity, specificity, area under the receiver operating characteristic curve (AUC), and Matthews Correlation Coefficient (MCC) (all %). Den201: DenseNet201; EffV2S: EfficientNetV2S; ViT: Vision Transformer-Base.

Model	F1-Score	Bal. Acc.	Precision	Recall	Sensitivity	Specificity	AUC	MCC
GNN-UNI & Eff.V2S	98.17 (±0.16)	98.14 (±0.16)	98.20 (±0.16)	98.14 (±0.16)	98.14 (±0.16)	98.14 (±0.16)	99.83 (±0.02)	96.35 (±0.33)
GNN-UNI2 & Eff.V2S	97.95 (±0.29)	97.94 (±0.28)	97.96 (±0.30)	97.94 (±0.28)	97.94 (±0.28)	97.94 (±0.28)	99.61 (±0.04)	95.90 (±0.58)
GNN-UNI & Den.201	98.24 (±0.36)	98.22 (±0.37)	98.26 (±0.34)	98.22 (±0.37)	98.22 (±0.37)	98.22 (±0.37)	99.86 (±0.02)	96.48 (±0.72)
GNN-UNI2 & Den.201	98.04 (±0.36)	98.03 (±0.36)	98.06 (±0.35)	98.03 (±0.36)	98.03 (±0.36)	98.03 (±0.36)	99.68 (±0.06)	96.09 (±0.72)

S2: Classification performance on the BACH dataset based on F1-score, balanced accuracy, precision, recall, sensitivity, specificity, area under the receiver operating characteristic curve (AUC), and Matthews Correlation Coefficient (MCC) (all %). Den201: DenseNet201; EffV2S: EfficientNetV2S; ViT: Vision Transformer-Base.

Model	F1-Score	Bal. Acc.	Precision	Recall	Sensitivity	Specificity	AUC	MCC
GNN-UNI & Eff.V2S	92.96 (±1.01)	93.00 (±1.00)	93.27 (±0.71)	93.00 (±1.00)	93.00 (±1.00)	97.66 (±0.33)	99.15 (±0.30)	90.77 (±1.24)
GNN-UNI2 & Eff.V2S	96.26 (±0.79)	96.25 (±0.79)	96.34 (±0.86)	96.25 (±0.79)	96.25 (±0.79)	98.75 (±0.26)	99.75 (±0.03)	95.02 (±1.07)
GNN-UNI & Den.201	93.22 (±1.00)	93.25 (±1.00)	93.52 (±0.89)	93.25 (±1.00)	93.25 (±1.00)	97.75 (±0.33)	99.03 (±0.25)	91.09 (±1.29)
GNN-UNI2 & Den.201	96.51 (±1.44)	96.50 (±1.45)	96.65 (±1.36)	96.50 (±1.45)	96.50 (±1.45)	98.83 (±0.48)	99.68 (±0.18)	95.37 (±1.92)

S3: Classification performance on the BreakHis dataset based on F1-score, balanced accuracy, precision, recall, sensitivity, specificity, area under the receiver operating characteristic curve (AUC), and Matthews Correlation Coefficient (MCC) (all %). Den201: DenseNet201; EffV2S: EfficientNetV2S; ViT: Vision Transformer-Base.

Model	F1-Score	Bal. Acc.	Precision	Recall	Sensitivity	Specificity	AUC	MCC
GNN-UNI & Eff.V2S	98.09 (±0.59)	97.68 (±0.85)	98.53 (±0.30)	97.68 (±0.85)	97.68 (±0.85)	97.68 (±0.85)	99.91 (±0.03)	96.21 (±1.16)
GNN-UNI2 & Eff.V2S	97.97 (±0.72)	97.05 (±1.03)	99.00 (±0.34)	97.05 (±1.03)	97.05 (±1.03)	97.05 (±1.03)	99.59 (±0.09)	96.03 (±1.40)
GNN-UNI & Den.201	98.09 (±0.59)	97.68 (±0.85)	98.53 (±0.30)	97.68 (±0.85)	97.68 (±0.85)	97.68 (±0.85)	99.90 (±0.03)	96.21 (±1.16)
GNN-UNI2 & Den.201	98.28 (±0.83)	97.50 (±1.19)	99.15 (±0.39)	97.50 (±1.19)	97.50 (±1.19)	97.50 (±1.19)	99.60 (±0.11)	96.63 (±1.60)
